# Surface phosphonation treatment shows dose-dependent enhancement of the bioactivity of polyetheretherketone

**DOI:** 10.1039/c9ra05229a

**Published:** 2019-09-23

**Authors:** Lvhua Liu, Yanyan Zheng, Qianyu Zhang, Lin Yu, Ziliang Hu, Ying Liu

**Affiliations:** School of Basic Medical Sciences, North Sichuan Medical College Nanchong China yanyzheng@163.com; Department of Stomatology, North Sichuan Medical College and Affiliated Hospital of North Sichuan Medical College Nanchong China ying_nsmc@hotmail.com; Department of Pharmacology, North Sichuan Medical College Nanchong China; Department of Preventive Medicine, North Sichuan Medical College Nanchong China

## Abstract

Polyetheretherketone (PEEK) is a promising alternative for biomedical metallic implants in orthopedic and dental applications because its elastic modulus is similar to that of bone. However, PEEK is a bioinert material that cannot be integrated with host bone. Our previous study showed surface phosphonation enhanced the osteogenic activity of PEEK. The purpose of this study was to evaluate the effect of the density of phosphonate groups on the bioactivity of PEEK. X-ray photoelectron spectroscopy and water contact angle measurement confirmed the successful grafting of different densities of phosphonate groups to the PEEK surface using a one-step ultraviolet-initiated graft polymerization method. Atomic force microscopy revealed that the surface treatment did not significantly alter the surface topography and roughness. *In vitro* biological evaluations showed that MC3T3-E1 osteoblast responses including adhesion, spreading, proliferation, alkaline phosphatase activity, extracellular matrix mineralization, collagen secretion, and osteogenesis-related gene expression exhibited dose-dependent enhancement depending on the density of phosphonate groups. Most importantly, histological analysis and biomechanical tests showed that in a rat femur implantation model, PEEK bearing phosphonate groups had a better bone-to-implant contact ratio and corresponding bone-to-implant bonding strength at 12 weeks post-implantation than unmodified PEEK. Thus, this work provides a simple method to boost the osteogenic activity and osseointegration ability of PEEK, which has potential clinical applications in orthopedic and dental implants.

## Abstract 2

Polyetheretherketone (PEEK) is a promising alternative for biomedical metallic implants in orthopedic and dental applications because its elastic modulus is similar to that of bone.

## Graphical abstract

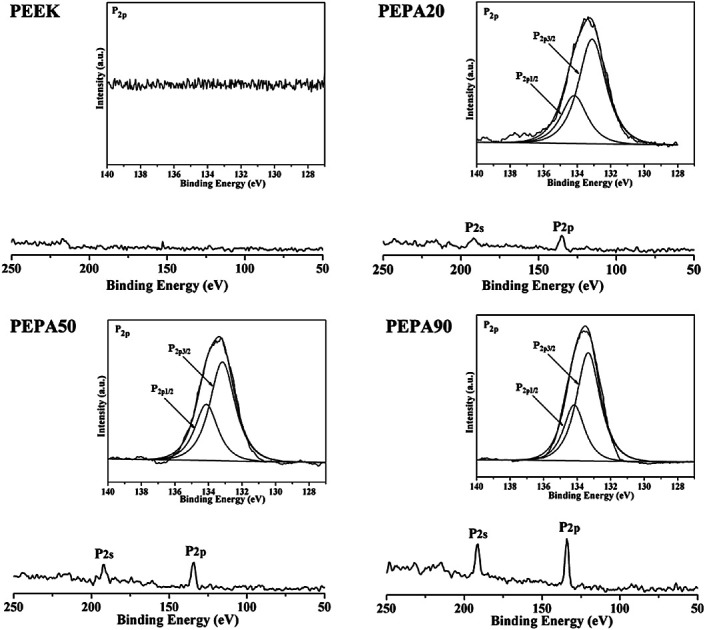

## 1. Introduction

Polyetheretherketone (PEEK) is a promising alternative to biomedical metal for orthopedic and dental implants due to its outstanding chemical resistance, satisfactory mechanical properties, and excellent sterilization resistance.^1,2^ Moreover, the elastic modulus of PEEK (3–4 GPa) is more analogous to that of human cortical bone (18 GPa) than that of titanium alloy (110 GPa). The modulus can be tailored to closely match that of cortical bone by preparing carbon-fiber reinforced composites, and thus the extent of stress shielding can be reduced or thoroughly eliminated. Additionally, its radiolucency facilitates the monitoring of bony fusion during the postoperative follow-up period.^3^ However, PEEK presents inferior osteogenic activity and bone-implant integration capability because of its bioinertness,^4,5^ and thus its clinical applications as orthopedic and dental implants are limited.

To address this deficiency, numerous research groups have exploited various strategies to tailor PEEK to enhance its osseointegration activity. One such strategy is to incorporate bioactive ceramics component including hydroxyapatite, calcium silicate and bioactive glass into the PEEK matrix to prepare a composite.^6–9^ However, the poor physical bonding between PEEK and ceramics results in a trade-off between mechanical properties and bioactivity. Alternatively, various techniques have been developed to deposit bioactive ceramics coatings on PEEK surfaces, such as microwave assisted coating,^10^ thermal plasma spray,^11^ spin coating,^12^ cold spray,^13^ ion beam assisted deposition,^14^ electron beam evaporation,^15^ and biomimetic.^16,17^ However, the ceramics coatings are apt to delaminate due to the weak bonding strength, and may induce severe inflammation and bone resorption.^18^ Additionally, layer-by-layer self-assembly and mussel-inspired surface chemistry strategies have been employed to prepare multi-functional PEEK surface with enhanced bacteriostasis and anti-inflammatory and osseointegrative properties.^19,20^ Surface chemistry is a pivotal factor in regard to cell and tissue response.^21,22^ As such, the tailoring of surface chemicals such as in the case of covalent grafting of functional groups or biomolecules without the hindrance of the detachment is a promising alternative to coating techniques. Our groups and others have reported that implants with incorporated functional groups such as carboxyl,^23,24^ sulfonic groups,^25^ amino^26^ and phosphonate groups,^27,28^ or biomolecules such as RGD peptides^29^ and antimicrobial peptides^30^ exhibit up-regulated bone cells osteogenic activity *in vitro* and osseointegration *in vivo*.

In comparison to other surface grafting approaches such as plasma- or radiation-induced grafting, photo-induced grafting has several advantages such as low cost, simple equipment, mild reaction conditions, easy of industrialization, weak penetrability, and long-term stability of the grafted chains without affecting the bulk materials.^31^ Recently, it has been shown that the diphenylketone moiety in the PEEK backbone acts as a photo-initiator, similar to that of benzophenone.^32^ When the PEEK surface is subjected to ultraviolet (UV) irradiation, semibenzopinacol-containing radicals of benzophenone units in the PEEK molecular structure can be self-induced, which initiates a polymerization reaction with functional monomers. As such, the PEEK surface can be modified with functional groups under UV-irradiation *via* a one-step and simple self-initiated graft polymerization method. The grafting of poly(acrylic acid), poly(2-(methacryloyloxy)ethyltrimethylammonium chloride) and poly(3-sulfopropyl methacrylate potassium salt) on PEEK surfaces has been achieved *via* photo-induced graft polymerization and the modified surfaces exhibited improved bio-tribological properties and aqueous lubrication.^33,34^ Our group has also used photo-induced graft polymerization to incorporate sulfonic groups and phosphonate groups on the surface of PEEK, which elevated the proliferation and osteogenic differentiation of osteoblast *in vitro*, and the bone-implant contact ratio *in vivo*.^25,28^

Previous studies have indicated that phosphonate groups enhance adhesion and differentiation of osteoblasts in a dose-dependent manner.^35,36^ Whereas, it is difficult to acquire the optimal content of phosphonate group because these investigations used copolymer containing other functional groups. In this investigation, different densities of phosphonate group were incorporated on a PEEK surface *via* a one-step UV-initiated graft polymerization method to screen out optimal surface-phosphorylated PEEK implants. The effects of surface phosphonation on the osteogenic activity of MC3T3-E1 osteoblast, including proliferation, mineralization, and osteogenic differentiation, were then examined. Moreover, *in vivo* osseointegration ability, including bone-implant contact ratio and bonding strength, was also assessed after implantation of the surface-phosphorylated PEEK into a rat's femur.

## 2. Materials and methods

### 2.1 Materials

Polyetheretherketone (PEEK) sheets were provided by Jiangsu Junhua High Performance Specialty Engineering Plastics (PEEK) Products Co., Ltd (Changzhou, China). The PEEK substrate was machined either into a disc format (*Φ*15 mm × 1 mm) for surface characterization and *in vitro* studies, or a plate format (6 mm × 2.8 mm × 1 mm) for *in vivo* animal experiments. Prior to use, all the samples were washed sequentially with 2-propanol, acetone, ethanol and ultrapure water in an ultrasonic cleaner, and finally dried under vacuum overnight. Vinylphosphonic acid (VPA) was provided by TCI (Shanghai) Development Co., Ltd. α-MEM, fetal bovine serum, and penicillin/streptomycin were purchased from Hyclone. Cell Counting Kit-8 (CCK-8) was provided by Dojindo (Kumamoto, Japan). BCA kit and ALP assay kit were purchased from Nanjing Jiancheng Bioengineering Institute. Alizarin Red, cetylpyridinium chloride, and Sirius Red were obtained from Sigma. TRIZOL reagent was from Invitrogen Life Technologies. PrimeScript RT reagent kit with gDNA Eraser and TB Green premix EX Taq II PCR kit were obtained from Takara (Dalian, China).

### 2.2 Preparation of surface-phosphorylated PEEK

Surface-phosphorylated PEEK was prepared as previously described.^28^ Briefly, PEEK specimens were immersed in 0.8 M VPA solution. A 1000 W high pressure mercury lamp (Beijing Institute of Electric Light Source) with a maximum intensity at approximately 365 nm was used to induce graft polymerization. The graft polymerization was performed for 20 min, 50 min and 90 min, and the corresponding poly(vinylphosphonic acid) (PVPA) grafted PEEK was abbreviated as PEPA20, PEPA50, and PEPA90, respectively. After the reaction, the PEPA samples were ultrasonically cleaned twice in ultrapure water for 20 min, to remove non-reacted monomers and non-grafted homopolymers on each occasion, and then subsequently dried at room temperature.

### 2.3 Surface characterization

The surface chemical composition of the substrates was investigated through X-ray photoelectron spectroscopy (XPS). XPS data were acquired using a Kratos Axis Ultra system with a monochromatized Al Kα source at a take off angle of 20°. The sample charging was calibrated to the reference of C1s set at 284.8 eV. Spectra data was analyzed using the analysis software XPS PEAK95 Version 3.1.

Surface wettability of the intact PEEK and PEPA was determined *via* static water contact angle measurements using the sessile drop method with a goniometer (Krüss DSA100, Germany). A volume of 3 μl ultra-pure water was placed on the surface of the samples using a motor driven syringe at room temperature. The mean value was calculated using four measurements.

The surface topography of PEEK samples was detected by atomic force microscopy (AFM; Dimension icon, Bruker, USA) in the tapping mode at room temperature. An area of 20 μm × 20 μm was imaged, and the roughness was calculated using the software NanoScope Analysis.

### 2.4 *In vitro* studies

#### 2.4.1 Culture of MC3T3-E1 cells

Mouse MC3T3-E1 osteoblasts were cultured in α-MEM with 10% fetal bovine serum, 1% penicillin/streptomycin in a humidified atmosphere of 5% CO_2_ at 37 °C. The medium was refreshed every three days. All the samples were sterilized using epoxy ethane before cell seeding.

#### 2.4.2 Morphological observation by scanning electron microscopy (SEM)

The MC3T3-E1 cells were seeded onto the samples at a density of 2.5 × 10^4^/well. After incubating for 4 h and 24 h, the medium was removed, and the samples were washed twice with sterile PBS and fixed with 2.5% glutaraldehyde overnight. Then, the fixed osteoblasts were progressively dehydrated with graded ethanol solutions (30%, 50%, 70%, 90%, 95% and 100%) for 15 min each. The samples were dried and sputter-coated with gold prior to SEM (Hitachi S-4200, Japan) observation of the osteoblast adhesion morphology.

#### 2.4.3 Cell attachment

The CCK-8 assay was used to determine the ability of the cultured osteoblast to adhere to the samples, as described elsewhere.^24^ Briefly, the cells were seeded on each sample with a density of 1.0 × 10^5^/well for 4 h. At a scheduled time, the specimens were rinsed three times with PBS and then incubated in 300 μl α-MEM with a supplement of 30 μl CCK-8 solution for 1.5 h. The solution was carefully transferred to a 96-well plate to measure the optical density using a microplate reader (SpectraMax M2, Molecular Devices, USA) at 450 nm.

#### 2.4.4 Cell proliferation

The cell proliferation was also evaluated using the CCK-8 assay. The cells were seeded on each sample with a density of 1.0 × 10^4^/well and cultured for 1, 4 and 7 days. The other detailed procedures were the same as those of the cell attachment test.

#### 2.4.5 Alkaline phosphatase (ALP) activity

Cells were seeded on different samples placed in 24-well plates with a density of 1 × 10^4^ cells per well for 7 and 14 days. At a prescribed time, the samples were washed thrice with PBS and cells were lysed using 1% Triton X-100 for 40 min at 4 °C. After centrifugation at 14 000 rpm for 10 min, the supernatant was extracted for total protein and ALP activity assay using a BCA kit and ALP assay kit following the manufacturer's respective protocols. The ALP activity of the test samples was normalized to the total protein content.

#### 2.4.6 Extracellular matrix (ECM) mineralization

After MC3T3-E1 cells were cultured on the samples for 7 and 14 days, the samples were washed thrice with PBS and fixed with 75% ethanol for 1 h. They were then stained with 40 mM Alizarin Red water solution (pH = 4.2) for 10 min at room temperature. Afterward, the samples were washed with distilled water until the orange color was no longer observed. For quantitative analysis, the adsorbed Alizarin Red was dissolved with 10% cetylpyridinium chloride in 10 mM sodium phosphate (pH = 7.0) and the absorbance was measured using a microplate reader at a wavelength of 620 nm.

#### 2.4.7 Collagen secretion

Collagen secretion of the MC3T3-E1 osteoblast on the specimens was quantified using Sirius Red staining. The cells were seeded on the samples with a density of 1.0 × 10^4^/well for 7 and 14 days. At a prescribed time, the samples were washed thrice with PBS and fixed in 4% paraformaldehyde. Following the three rinses in PBS, the samples were stained with a 0.1% solution of Sirius Red in saturated picric acid for 18 h. They were then washed with 0.1 M acetic acid until the red color was not observed. For quantitative analysis, the stain on the samples was dissolved using 500 μl eluent (0.2 M NaOH/methanol = 1 : 1). The absorbance at 540 nm was then measured on a microplate reader.

#### 2.4.8 Real-time quantitative PCR (RT-PCR) analysis

The expression of osteogenesis-related genes, including alkaline phosphatase (ALP), osteocalcin (OCN), collagen type I (COL-I), and osteopontin (OPN) were quantitatively analyzed by RT-PCR using β-actin as the housekeeping gene for normalization. The sequences of the forward and reverse primers are listed in Table 1. The total RNA was collected from cells grown on the samples using TRIZOL reagent according to the manufacturer's instructions. A total of 1 μg RNA was reverse transcribed using the PrimeScript RT reagent kit with gDNA Eraser. RT-PCR (CFX Connect, Bio-Rad, USA) was performed using the TB Green premix EX Taq II PCR kit.

**Table 1:** Primer pairs used in real-time analysis

Gene	Primers (F = forward, R = reverse)
ALP	F: CTCCATCTTTGGTCTGGCTCC
	R: CCTGGTAGTTGTTGTGAGCGTAAT
OCN	F: TGGCTGCGCTCTGTCTCTCT
	R: TTCACTACCTTATTGCCCTCCTG
COL-I	F: CTGGACGCCATCAAGGTCTACT
	R: AACGGGAATCCATCGGTCAT
OPN	F: TAGGAGTTTCCAGGTTTCTGATGA
	R: CTGCCCTTTCCGTTGTTGTC
β-Actin	F: AGATTACTGCTCTGGCTCCTAGC
	R: ACTCATCGTACTCCTGCTTGCT

### 2.5 *In vivo* studies

#### 2.5.1 Surgical procedures

All animal procedures and experiments were performed according to the guidelines of the Institutional Animal Care and Use Committee of China. Male Sprague-Dawley rats (200 ± 20 g) were used with the approval of the Institutional Animal Care and Treatment Committee of North Sichuan Medical College. A rat femur distal model was used for the *in vivo* osseointegration study. Prior to surgery, the rats were anesthetized by intraperitoneal injection of sodium pentobarbital solution (50 mg kg^−1^), and the medial side of the knees of both legs was shaved and depilated. Afterwards, a longitudinal incision of approximately 15 mm long was made down to the periosteum. A slit-like incision (2.8 mm × 1 mm) was prepared at the left and right distal tibiae parallel to the long axis of this bone using a dental drill. Subsequently, the implants were inserted into the slit in a press-fit manner. Finally, the wound was carefully closed layer-by-layer. After the operation, the rats received a subcutaneous injection of oxytetracycline (30 mg kg^−1^) for 3 days. They were euthanized 12 weeks postsurgery, and the femur bones containing the PEEK implants were retrieved and fixed in 10% formaldehyde.

#### 2.5.2 Histological evaluation

The samples were dehydrated using increasing concentrations of ethanol, and embedded in polymethylmethacrylate resin. The embedded specimens were cut into 50 μm thick sections perpendicular to the femur axis using a Leica SP1600 saw microtome (Leica, Hamburg, Germany). The sectioned samples were stained using toluidine blue-fuchsine, and the integration of the bone-implant was observed using optical microscopy (Olympus, Wild Mp5, Japan). Histomorphometric analysis was performed *via* evaluation of bone-implant contact (BIC) ratios based on the acquired images using the Image-Pro Plus 6.0 software.

#### 2.5.3 Biomechanical testing

To investigate the bone-implant interface bonding, pull-out tests were performed on a biomechanical apparatus under a displacement speed of 1 mm min^−1^. The pull-out load was calculated by averaging the results from five pull-out tests.

### 2.6 Statistical analysis

All experiments were performed at least in triplicate. One-way analysis of variance (ANOVA), Tukey's multiple comparison tests for *in vitro* evaluation, and paired *t*-test for *in vivo* evaluation was used to determine the statistical significance of observed differences using SPSS 16.0 statistical software. *p* < 0.05 was considered statistically significant.

## 3. Results

### 3.1 Surface characterization

**Fig. 1:**
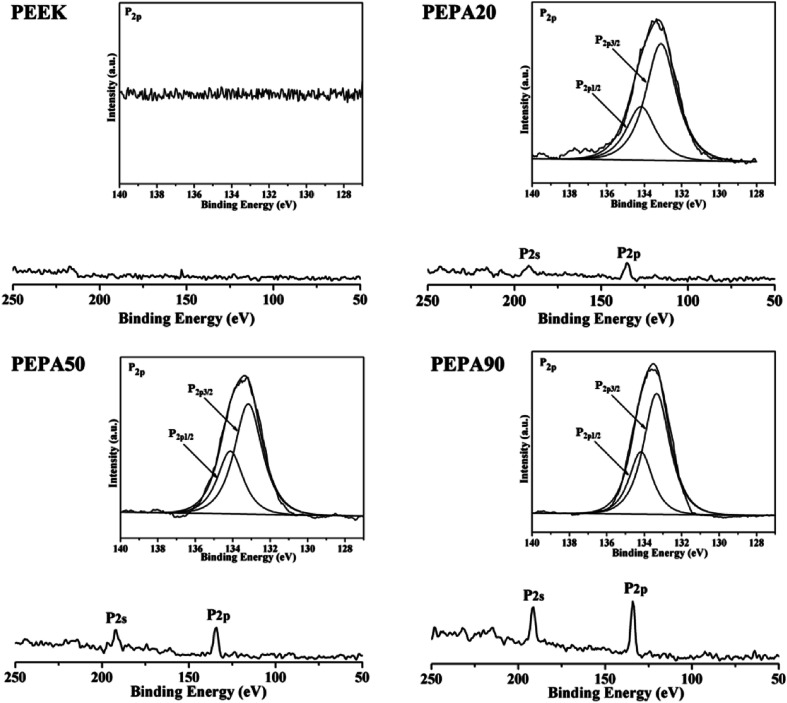
XPS full and P2p high-resolution spectra obtained from PEEK, PEPA20, PEPA50, and PEPA90 samples.

**Fig. 2:**
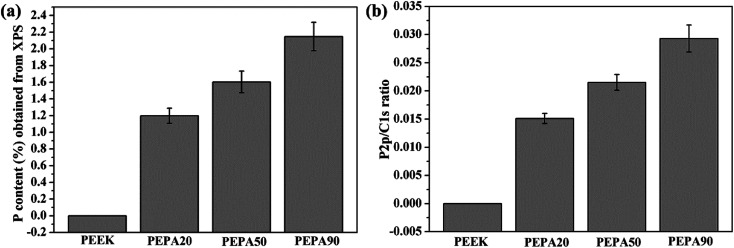
(a) P concentration in atomic percent and (b) P2p/C1s ratio obtained from XPS results for PEEK, PEPA20, PEPA50, and PEPA90 surfaces.

To confirm the successful grafting polymerization of VPA on the PEEK substrate, PEEK, PEPA20, PEPA50, and PEPA90 samples were analyzed using XPS. Fig. 1 displays the XPS full and P2p high-resolution spectra of unmodified and surface-treated PEEK. The XPS full spectra revealed that the characteristic peak of P2p and P2s were detected in the PEPA20, PEPA50, and PEPA90 samples. Furthermore, the high-resolution P2p spectrum for PEPA20, PEPA50 and PEPA90 exhibited two major peaks at 133.3 eV and 134.1 eV associated with P2p_3/2_ and P2p_1/2_ that originated from high oxidation state phosphorus, *i.e.* phosphate groups.^37^ Thus, the XPS results indicate that phosphonate groups were successfully introduced on the PEEK surface. The atomic percent of P and P2p/C1s ratio were quantified using the XPS survey scans and the results are depicted in Fig. 2. Clearly, there is a progressive increase in the concentration of the phosphonate group from 1.19% to 2.15% as the photo-induced graft polymerization time increases from 20 min to 90 min (Fig. 2a). Additionally, the steady increase in the P2p/C1s ratio (Fig. 2b) is indicative of the increase of the content of the phosphonate group on the PEEK surface.

**Fig. 3:**
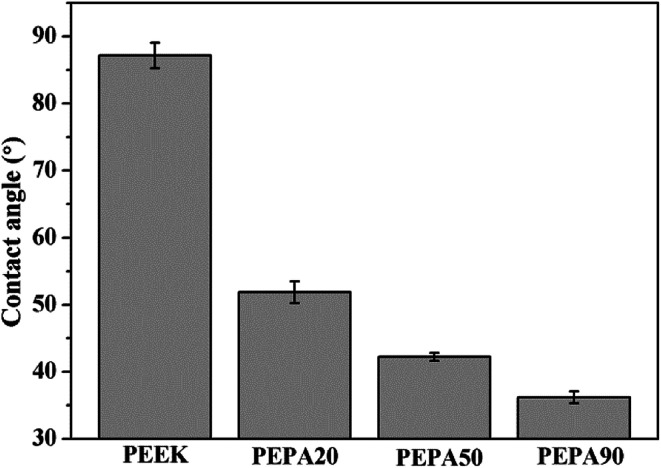
Histogram of static water contact angles of PEEK, PEPA20, PEPA50, and PEPA90 substrates.

After verification of the successful chemical modifications *via* XPS, the static water contact angles of all the PEEK samples were measured and the results are summarized in Fig. 3. The water contact angle of PEEK is 87.2°, while the contact angle decreases to 51.8° for PEPA20, 42.2° for PEPA50 and 36.2° for PEPA90. This indicates that the surface becomes more hydrophilic after the introduction of phosphonate groups on the PEEK surface. Additionally, the decreasing contact angle is indirectly indicative of the gradually increasing content of the phosphonate groups on the PEEK surface.

**Fig. 4:**
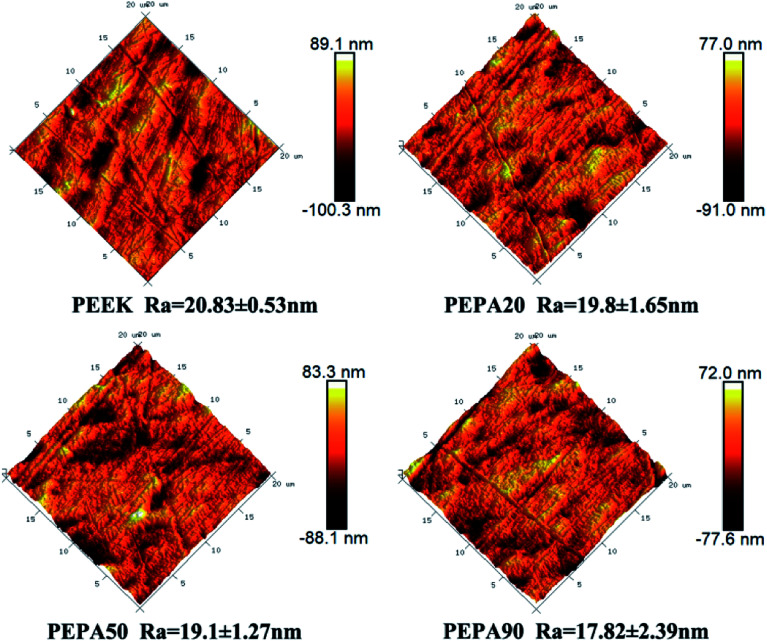
AFM images and the corresponding *R*_a_ roughness of PEEK, PEPA20, PEPA50, and PEPA90 samples.

The surface morphologies of PEEK, PEPA20, PEPA50, and PEPA90 substrates were examined *via* AFM. AFM images of all PEEK substrates and the corresponding *R*_a_ roughness are shown in Fig. 4. Pristine and surface-treated PEEK exhibits a similar topography and the *R*_a_ roughness is in the range of 17 to 20 nm. This indicates that surface graft polymerization does not alter the surface topography and roughness.

### 3.2 Responses of MC3T3-E1 *in vitro*

#### 3.2.1 Cell adhesion and cell proliferation

**Fig. 5:**
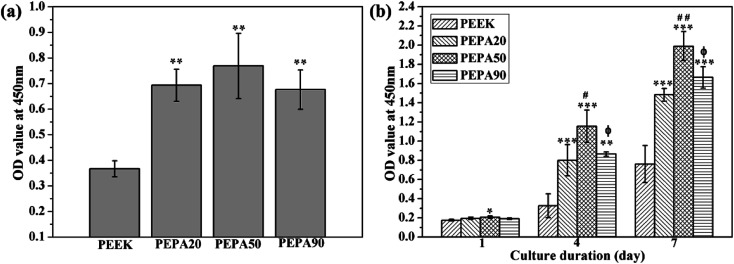
(a) Cell adhesion and (b) proliferation of MC3T3-E1 osteoblasts after culturing for 4 h, 1, 4, and 7 days as determined by CCK-8. *(*p* < 0.05), **(*p* < 0.01), and ***(*p* < 0.001) when compared to those of PEEK; ^#^(*p* < 0.05), and ^##^(*p* < 0.01) when compared to those of PEPA20; and ^ϕ^(*p* < 0.05) when compared to that of PEPA50.

Fig. 5a presents the quantitative analysis of cell adhesion on intact and surface-phosphorylated PEEK substrates surfaces. After 4 h of culture, more MC3T3-E1 cells were observed to attach on the surface-phosphorylated PEEK than on PEEK (*p* < 0.01), indicating that surface phosphonation could enhance the attachment of MC3T3-E1 cells. Additionally, the adherent cell numbers on PEPA50 were higher than those of PEPA20 and PEPA90. However, cells adherent on PEPA50 exhibited no statistical difference compared to those of cells adherent on PEPA20 and PEPA90.

The time-related proliferation of MC3T3-E1 cells cultured on all PEEK substrates surfaces was measured using CCK-8 assay as shown in Fig. 5b. After culturing for 1 day, the proliferation of MC3T3-E1 cells on PEPA50 was statistically higher (*p* < 0.05) than that on PEEK, whereas this was not the case for PEPA20 and PEPA90. When the culturing time was extended to 4 and 7 days, the number of MC3T3-E1 cells on the surface-phosphorylated PEEK substrates was significantly higher than that on PEEK (*p* < 0.001). This indicates that surface phosphonation is more beneficial to MC3T3-E1 cells proliferation and causes no cytotoxic effects on cells. Additionally, the proliferation activity of MC3T3-E1 cells grown on PEPA50 was significantly higher (*p* < 0.05) than that of the cells grown on PEPA20 and PEPA90.

#### 3.2.2 Cell morphology and spreading

**Fig. 6:**
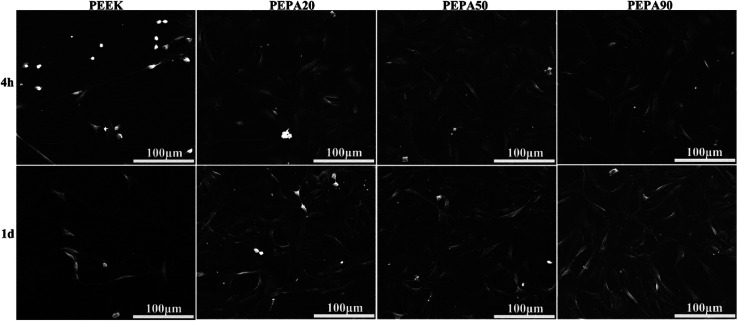
SEM morphology of the MC3T3-E1 on the samples after culturing for 4, and 24 h.

The morphology of the MC3T3-E1 cells grown on all the PEEK substrates surfaces at different time points was observed by SEM and the images are presented in Fig. 6. After culturing for 4 h, the majority of MC3T3-E1 cells on the PEEK surface present a spherical morphology with few filopodia, even though a few cells exhibited an elongated morphology. However, the cells on the surface-phosphorylated PEEK surfaces extend their cytoplasmic to the entire surface with many filopodia and lamellipodia. After culturing for 24 h, the MC3T3-E1 cells attach and spread well on all the PEEK substrates surfaces. In addition, the cells stacked and stretched more, and they were more interconnected with each other through longer filopodia and lamellipodia on the surface-phosphorylated PEEK.

#### 3.2.3 ALP activity, extracellular matrix mineralization and collagen secretion

**Fig. 7:**
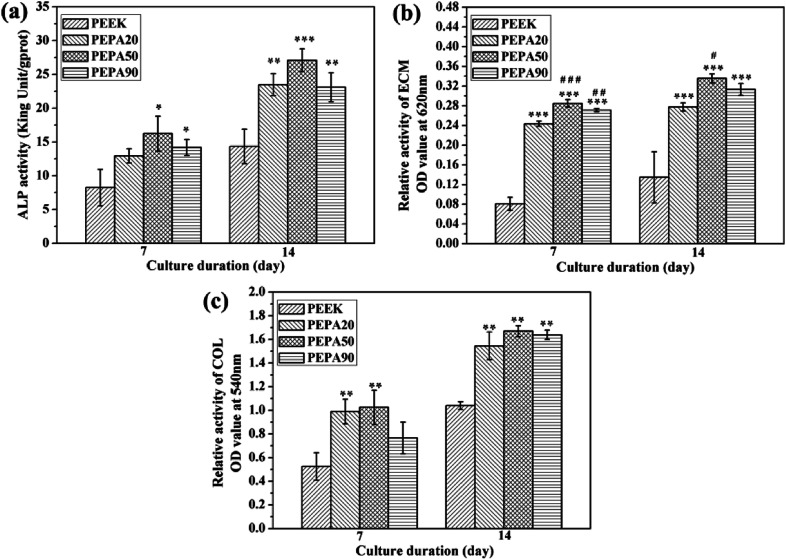
(a) ALP activity, (b) ECM mineralization assay and (c) COL secretion assay of MC3T3-E1 osteoblasts after culturing for 7 and 14 days. *(*p* < 0.05), **(*p* < 0.01), and ***(*p* < 0.001) when compared to those of PEEK; ^#^(*p* < 0.05), and ^##^(*p* < 0.01) when compared to those of PEPA20.

To evaluate the effect of surface phosphonation on the osteogenic differentiation of MC3T3-E1 osteoblasts, the ALP activity of the cells on all the PEEK substrate surfaces was measured after culturing for 7 and 14 days. As shown in Fig. 7a, the ALP activity of the MC3T3-E1 osteoblasts cultured on surface-phosphorylated PEEK was significantly higher (*p* < 0.05) than that of the unmodified PEEK. This indicates that surface phosphonation can improve the initial osteogenic differentiation of MC3T3-E1 osteoblasts grown on PEEK. Additionally, MC3T3-E1 osteoblasts cultured on PEPA50 displayed a relatively higher ALP activity compared to those of osteoblasts cultured on PEPA20 and PEPA90, although the difference between them was not significant.

The quantitative results for the ECM mineralization of MC3T3-E1 osteoblasts cultured on all PEEK substrates surfaces for 7 and 14 days are depicted in Fig. 7b. The results show that ECM mineralization levels of MC3T3-E1 osteoblasts grown on surface-phosphorylated PEEK are significantly up-regulated (*p* < 0.05) compared to those grown on the PEEK control. MC3T3-E1 osteoblasts cultured on PEPA20 and PEPA90 exhibited comparable ECM mineralization levels during the observation period. Moreover, ECM mineralization of MC3T3-E1 osteoblasts seeded on PEPA50 was higher compared to those on the PEPA20 and PEPA90 samples. Moreover, ECM mineralization of MC3T3-E1 osteoblasts seeded on PEPA50 was significantly higher (*p* < 0.05) compared to that on PEPA20.

The quantitative results for collagen secretion of MC3T3-E1 osteoblasts cultured on all the PEEK substrates are displayed in Fig. 7c. Collagen secretion of MC3T3-E1 osteoblasts cultured for 7 and 14 days on surface-phosphorylated PEEK samples improved significantly (*p* < 0.05) compared to that on the PEEK control. In addition, MC3T3-E1 osteoblasts grown on the surface-phosphorylated PEEK exhibited a comparable collagen secretion level throughout the observation period.

#### 3.2.4 Expression of osteogenic differentiation-related genes

**Fig. 8:**
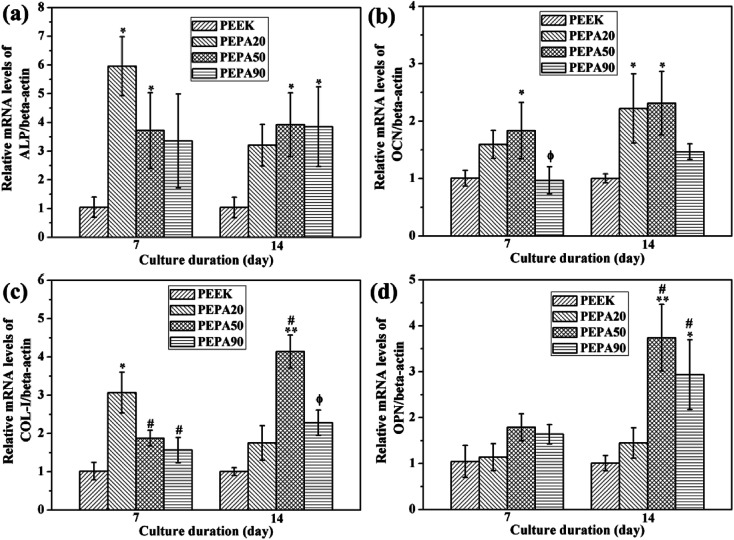
Relative mRNA expression of osteogenesis-related genes in MC3T3-E1 cells grown on the samples measured by RT-PCR: (a) ALP, (b) OCN, (c) COL-I, and (d) OPN. *(*p* < 0.05) and **(*p* < 0.01) when compared to those of PEEK; ^#^(*p* < 0.05) when compared to that of PEPA20; ^ϕ^(*p* < 0.05) when compared to that of PEPA50.

Fig. 8 displays the results of RT-PCR of the osteogenesis-related genes. In general, MC3T3-E1 cells grown on the surface-phosphorylated PEEK showed obviously higher expression levels of ALP, OCN, COL-I and OPN compared to those grown on the PEEK control during the culture period. Additionally, the COL-I and OPN mRNA levels of MC3T3-E1 cells cultured on PEPA50 were significantly higher (*p* < 0.05) than those grown on the PEPA20 at day 14. In addition, MC3T3-E1 cells cultured on PEPA50 exhibited obviously higher expression levels of OCN compared to those grown on PEPA90 at day 7 and 14.

### 3.3 *In vivo* studies

**Fig. 9:**
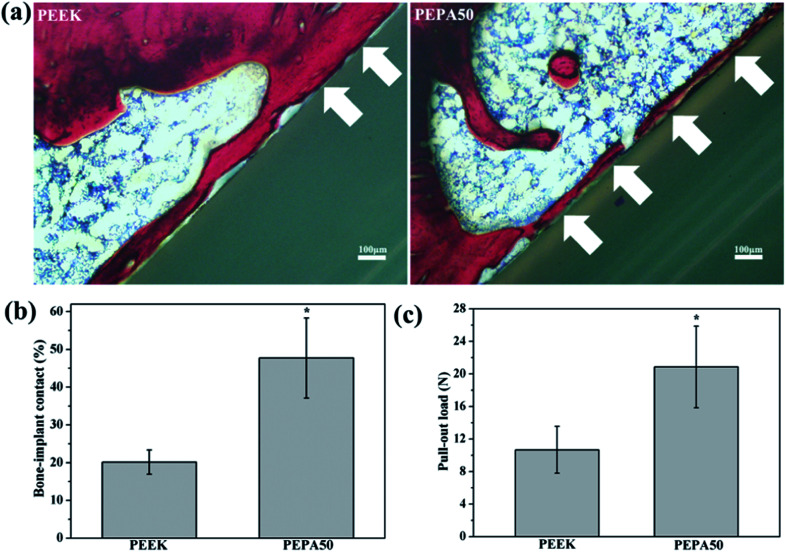
(a) Hard tissue sections of toluidine blue-fuchsine stained around the implant at 12 weeks post-implantation with the white arrows marking the direct bone contact between bone tissue and PEEK substrates. (b) Percentage of bone-implant contact ratios and (c) pull-out load between bone tissue and the PEEK samples after implantation for 12 week. *(*p* < 0.05) when compared to that of PEEK.

The aforementioned results demonstrate that surface phosphonation affects osteoblast activity in a dose-dependent manner, and thus PEPA50 with an optimum phosphonate group concentration was used for the *in vivo* implantation studies. To compare osseointegration capability, PEEK and PEPA50 were implanted into rat femurs, and the bone-implant contact ratio (Fig. 9a and b) and bone-implant bonding strength (Fig. 9c) were determined at 12 weeks post-implantation. PEPA50 exhibited a significantly higher (*p* < 0.05) bone-implant contact ratio than PEEK. In accordance with this result, the bone-implant bonding strength was significantly higher (*p* < 0.05) in PEPA50 compared to that in PEEK. These results demonstrate that surface phosphonation improves the osseointegration activity of PEEK.

## 4. Discussion

Although the resonance stabilized chemical structure of PEEK confers its outstanding chemical resistance and biocompatibility, the structure also makes it extremely difficult to functionalize PEEK *via* chemical reactions. Nevertheless, many scholars have pursued some exploratory work using wet chemistry.^16,23,29,37,38^ A modified PEEK surface *via* wet chemistry has been reported to improve the bioactivity of PEEK with respect to the enhancement of the bone-implant interface. However, there are several disadvantages such as multi-step procedures and time-consuming reactions. Fortunately, the benzophenone groups in the PEEK backbone generates active radical on its surface under UV-irradiation. And the radicals directly initiate functional monomer polymerization and surface-functionalized PEEK can be obtained in a relatively short time period. In this case, PEEK surfaces with different content of phosphonate groups were prepared *via* UV-initiated graft polymerization to identify optimal surface-phosphorylated PEEK implants based on previous reports where it was shown that phosphate modification ameliorated the bioactivity and osseointegration capability of PEEK implants both *in vitro* and *in vivo*.^23,28,37^

Cell and tissue responses can be modulated by altering the surface properties of biomaterials such as the surface chemical composition, surface topography and roughness.^39–41^ In this work, XPS confirmed that different amounts of phosphonate groups are incorporated onto the PEEK surface *via* one-step UV-initiated graft polymerization (Fig. 1 and 2). It is worth noting that no obvious surface topography and roughness changes were observed after surface phosphonation (Fig. 4). Thus, the impact of surface topography and roughness on biological responses can be excluded.

To evaluate the influence of surface phosphonation on the osteointegration of PEEK, both *in vitro* and *in vivo* studies are conducted. Adhesion, spreading and proliferation of MC3T3-E1 osteoblasts improved after surface phosphonation (Fig. 5 and 6) and the good cytocompatibility of poly(vinylphosphonic acid) was also confirmed. ALP activity, ECM mineralization, COL secretion, and expressions of osteogenic differentiation-related genes of MC3T3-E1 osteoblasts were quantitatively measured to evaluate osteogenic differentiation of MC3T3-E1 osteoblasts *in vitro*. In general, MC3T3-E1 osteoblasts on the phosphorylated surfaces exhibit enhanced osteogenic differentiation compared to that on PEEK control (Fig. 7 and 8). Taken together, PEPA50 shows the optimal MC3T3-E1 osteoblasts adhesion, growth and osteogenic differentiation. Similar findings were observed by other research groups. Dadsetan *et al.* observed that oligo(polyethylene glycol) fumarate hydrogel containing 620 μmol bis(2-(methacryloyloxy)ethyl)phosphate showed the optimal human fetal osteoblast proliferation and ALP activity.^35^ Gemeinhart *et al.* created a phosphonate-containing copolymer-modified surface and observed that surface modified with 30% vinyl phosphonic acid in the feed exhibited a maximal cell adhesion, proliferation, and calcification.^36^ Some explanations can be found in the literature to explain why surface phosphonation influences osteoblast activity. Phosphonate groups are negatively charged under physiological conditions, and thus are capable of chelating calcium ions from media, which is a propitious feature for transducing osteogenic cues.^42,43^ In fact, polymers and metal bearing phosphonate groups initially attract calcium ions and then induce bone-like apatite deposition on their surface.^16,44,45^ Then the polymers and metal directly bond to living bone *via* the bone-like apatite layer. Recently, accumulating evidence suggests that bone regeneration is strongly influenced by the cross-talk between bone-forming cells and immune cells, particularly macrophages.^46,47^ An efficient and timely switch from pro-inflammatory M1 to anti-inflammatory M2 macrophage phenotype results in an osteogenic cytokine release and the corresponding formation of new bone tissue.^46^ In addition, the physicochemical properties of the materials play prominent roles in macrophage plasticity. A recent study demonstrated that more macrophages might be polarized toward an M2 macrophage phenotype on surface-phosphorylated PEEK than pristine PEEK.^27^ Additionally, it has been reported that the surface chemistry was able to influence the type, quantity and activity of adsorbed matrix proteins, including vitronectin, collagen I, fibronectin and laminin.^48^ In addition, osteoblast adhesion, spreading, proliferation and differentiation have been reported to depend on these proteins. A study by Tan *et al.*, demonstrated that protein adsorption peaked in a dose-dependent manner during the investigation of protein adsorption on substrates containing phosphonate groups.^49^ As the concentration of the phosphonate group on the substrate surface exceeds a specific threshold, there is greater repulsion between the substrate and the negatively charged proteins, which consequently leads to a decrease in protein adsorption and a decrease in cell response.^50^ Therefore, MC3T3-E1 osteoblasts on the PEPA50 surface exhibit optimal overall responses in terms of adhesion, spreading, proliferation and osteogenic differentiation. These reasons partially account for the excellent osteogenic activity of MC3T3-E1 osteoblasts observed on the surface-phosphorylated PEEK, however, the underlying mechanism remains to be further elucidated.

Finally, we compared the osseointegration capabilities of PEEK and PEPA50 implants in terms of the bone-implant contact ratio and bonding strength using the rat femur implantation model. In this study, we determined that the surface-phosphorylated PEEK exhibited significantly higher bone-implant contact ratios (Fig. 9a and b) and bone-implant bonding strength (Fig. 9c) compared to those of the pristine PEEK at 12 weeks after implantation. In this case, we demonstrated that PEEK surface bearing phosphonate groups enhanced osteoblast responses such as proliferation, alkaline phosphatase activity, extracellular matrix mineralization, collagen secretion, and osteogenesis-related genes expression. These data suggest that surface-phosphorylated PEEK provides a more favorable microenvironment for bone cells adhesion, proliferation, and differentiation, and thus facilitates enhanced osseointegration capability.

## 5. Conclusions

PEEK surface bearing various phosphonate groups content was fabricated *via* a one-step UV-initiated graft polymerization of vinyl phosphonic acid. The *in vitro* osteoblast response results indicate that phosphonate groups are competent to enhance cell adhesion, cell spreading, cell proliferation, alkaline phosphatase activity, extracellular matrix mineralization, collagen secretion, and osteogenesis-related genes expression of MC3T3-E1 osteoblast in a dose-dependent manner. And the surface-phosphorylated PEEK generated after 50 min of UV induced grafting shows the optimal osteoblasts responses. Moreover, the *in vivo* results show that the surface-phosphorylated PEEK is more beneficial for bone tissue growth and osseointegration than intact PEEK surfaces. It was determined that the surface-phosphorylated PEEK provides a more favorable surface for bone regeneration compared to that of the bare PEEK surface, which boosts the potential for future clinical applications as orthopedic and dental implants.

## Conflicts of interest

There are no conflicts to declare.

## Supplementary Material
