# Correction: Surface phosphonation treatment shows dose-dependent enhancement of the bioactivity of polyetheretherketone

**DOI:** 10.1039/d0ra90045a

**Published:** 2020-04-30

**Authors:** Lvhua Liu, Yanyan Zheng, Qianyu Zhang, Lin Yu, Ziliang Hu, Ying Liu

**Affiliations:** School of Basic Medical Sciences, North Sichuan Medical College Nanchong China yanyzheng@163.com; Department of Stomatology, North Sichuan Medical College, Affiliated Hospital of North Sichuan Medical College Nanchong China ying_nsmc@hotmail.com; Department of Pharmacology, North Sichuan Medical College Nanchong China; Department of Preventive Medicine, North Sichuan Medical College Nanchong China

## Abstract

Correction for ‘Surface phosphonation treatment shows dose-dependent enhancement of the bioactivity of polyetheretherketone’ by Lvhua Liu *et al.*, *RSC Adv.*, 2019, **9**, 30076–30086, DOI: 10.1039/C9RA05229A.

The authors regret that an incorrect version of Fig. 9 was included in the original article. The correct version of Fig. 9 is presented below.

**Fig. 9:**
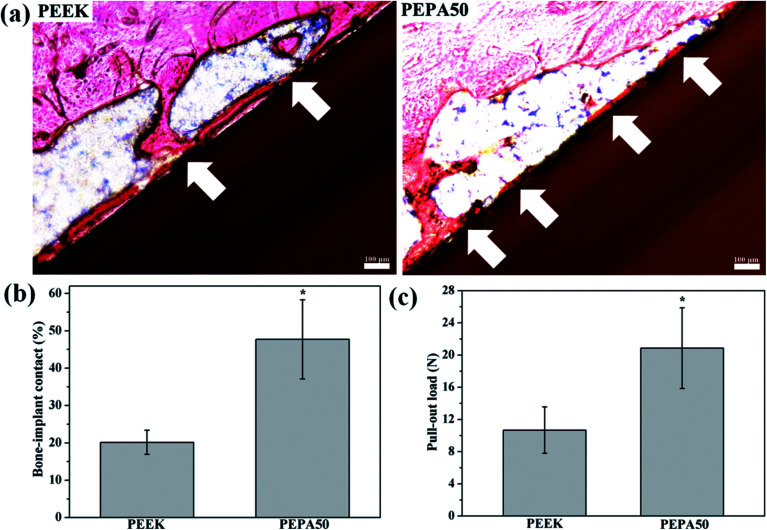
(a) Hard tissue sections of toluidine blue-fuchsine stained around the implant at 12 weeks post-implantation with the white arrows marking the direct bone contact between bone tissue and PEEK substrates. (b) Percentage of bone-implant contact ratios and (c) pull-out load between bone tissue and the PEEK samples after implantation for 12 weeks; *(*p* < 0.05) when compared to that of PEEK.

The Royal Society of Chemistry apologises for these errors and any consequent inconvenience to authors and readers.

## Supplementary Material

